# Transient Neonatal Diabetes Mellitus Potentially Associated With a Novel Homozygous MS4A6A Gene Variant: A Case Report

**DOI:** 10.7759/cureus.101561

**Published:** 2026-01-14

**Authors:** Sohrab Shakeel, Sandeep Kadam, Sameer Pawar, Dhyey Pandya, Pragathi Kamath, Rahul Dawre, Kanchan Sakharkar, Abhinav Kachare, Sangeeta Chivale, Suvidha Sardar, Abhilash Yamavaram, Poonam Mane, Prakash Gambhir, Parag M Tamhankar, Salil Vaniawala, Aarti A Kinikar

**Affiliations:** 1 Neonatology, Byramjee Jeejeebhoy Government Medical College, Pune, IND; 2 Neonatology, KEM Hospital, Pune, IND; 3 Pediatrics, Byramjee Jeejeebhoy Government Medical College and Sassoon General Hospital, Pune, IND; 4 Pediatrics and Child Health, Byramjee Jeejeebhoy Government Medical College, Pune, IND; 5 Pediatrics and Genetics, Byramjee Jeejeebhoy Government Medical College, Pune, IND; 6 Genetics, SN GeneLab Pvt Ltd, Surat, IND; 7 Genetics, Center for Medical Genetics, Mumbai, IND

**Keywords:** monogenic diabetes, ms4a6a, neonatal diabetes, transient ndm, whole exome sequencing

## Abstract

Neonatal diabetes mellitus (NDM) is a rare metabolic disorder characterised by hyperglycemia within the first six months of life. While commonly monogenic, the *MS4A6A* gene, known for immune modulation and calcium signalling, has not previously been linked to NDM. We report a case of transient NDM (TNDM) potentially associated with a novel homozygous variant in the *MS4A6A* gene. A male infant born at 27 weeks of gestation (720 g) developed severe hyperglycemia (>200 mg/dL) and polyuria on day 14 of life, following the resolution of suspected meningitis. Investigations confirmed insulin-deficient diabetes with low C-peptide (0.3 ng/mL) and negative antibodies. While standard NDM genetic panels were negative, whole-exome sequencing identified a homozygous *MS4A6A* variant (c.162G>C; p.Leu54Phe). Chromosomal microarray confirmed a region of loss of heterozygosity (LOH) at 11q12.1q12.2, encompassing the *MS4A6A* gene, consistent with identity by descent. The variant is extremely rare in population databases and is predicted to be damaging by multiple *in silico* tools. Both asymptomatic parents were confirmed as heterozygous carriers. The infant was treated with insulin, achieving excellent catch-up growth. However, by day 92, insulin requirements decreased, and the patient spontaneously maintained euglycemia off therapy by day 95. Remission was biochemically confirmed by normalised serum insulin levels (3.97 μIU/mL). This clinical course suggests a potential mechanism involving transient islet inflammation or defective calcium signalling. This case identifies *MS4A6A* as a potential candidate gene for TNDM, necessitating lifelong surveillance for relapse and highlighting a "dual-risk" profile for the heterozygous parents.

## Introduction

Neonatal diabetes mellitus (NDM) is a rare metabolic disorder characterised by hyperglycemia beginning within the first six months of life [1]. With an estimated incidence of one in 90,000-160,000 live births [2], it is broadly classified into two main groups: permanent neonatal diabetes (PNDM), which requires lifelong treatment, and transient neonatal diabetes mellitus (TNDM), where hyperglycemia spontaneously resolves, typically within the first few months, but carries a high risk of relapse in later life [3]. While PNDM is genetically heterogeneous [4,5], TNDM is most commonly associated with abnormalities at the 6q24 locus [3,6]. The etiology of NDM is predominantly monogenic, with mutations in the K-ATP channel genes (*KCNJ11*,* ABCC8*) and the insulin (*INS*) gene being the most common overall [7,8]. A precise genetic diagnosis is paramount, as it informs prognosis and therapy [9].

Despite these advances, a subset of NDM cases remains without a molecular diagnosis. The *MS4A6A* gene is part of the membrane-spanning 4-domains, subfamily A (MS4A) group, known for its role in immune modulation and function as a calcium-permeable ion channel [10]. Unlike typical NDM genes involved in beta-cell embryogenesis, *MS4A6A* is predominantly expressed in myeloid cells and is implicated in inflammatory regulation [10]. Herein, we present the first case of TNDM potentially associated with a homozygous variant in the *MS4A6A* gene. Trio-based sequencing and chromosomal microarray confirmed an autosomal recessive inheritance pattern driven by identity by descent (IBD) [11]. This report describes a potential new monogenic association for NDM, provides in silico and chromosomal evidence of pathogenicity, and proposes a novel pathophysiological mechanism involving transient inflammation and calcium flux dysregulation.

## Case presentation

A male infant born at 27 weeks and six days of gestation (birth weight 720 g) was admitted to the neonatal intensive care unit (NICU). He was born to asymptomatic, non-consanguineous parents, though a significant polygenic risk for diabetes was noted in the family history. His early course was complicated by respiratory distress requiring intubation, Grade II intraventricular haemorrhage, and suspected meningitis. The infant was treated with a 21-day course of intravenous antibiotics, specifically meropenem and gentamycin.

Around day 10 of life, the infant developed polyuria and weight loss. On day 14, persistent hyperglycemia (>200 mg/dL) (Table 1) was noted. To rule out sepsis-induced stress hyperglycemia, a full septic workup was performed. Blood cultures were sterile, and the C-reactive protein (CRP) was 2 mg/L (normal <3 mg/L) (Table 1), effectively ruling out active infection as the primary cause. Additionally, arterial blood gas (ABG) analysis showed a pH of 7.42 and bicarbonate (HCO_3_) of 22 mmol/L (Table 1), ruling out diabetic ketoacidosis (DKA).

**Table 1:** Summary of key investigations LOH: loss of heterozygosity; CMA: chromosomal microarray; ABG: arterial blood gas; CRP: C-reactive protein; DKA: diabetic ketoacidosis; VUS: variant of uncertain significance

Parameter	Result	Reference Range	Interpretation
Blood Glucose	260–320 mg/dL	60–120 mg/dL	Hyperglycemia
Serum Insulin (Day 14)	1.2 μIU/mL	2–20 μIU/mL	Low (Insulin Deficiency)
β-Hydroxybutyrate	4.1 mmol/L	<0.6 mmol/L	High (Ketosis)
C-Peptide	0.3 ng/mL	0.9–7.1 ng/mL	Low
CRP	2 mg/L	<3 mg/L	Sepsis ruled out
Blood Culture	Negative	—	Sterile
ABG (pH/HCO3)	7.42/22 mmol/L	7.35-7.45/22-26	No Acidosis (No DKA)
Microarray (CMA)	arr[hg19] 11q12.1q12.2 (59,014,657-61,457,857) hmz	Negative for Del/Dup	LOH/Identity by Descent
WES (Proband)	MS4A6A variant	Homozygous	Variant identified (VUS)
WES (Parents)	MS4A6A variant	Heterozygous	Carrier of variant
Serum Insulin (Day 97)	3.97 μIU/mL	2–20 μIU/mL	Normal (Restoration of Secretion)

Endocrine investigations confirmed insulin-deficient diabetes with low serum insulin (1.2 μIU/mL) and C-peptide (0.3 ng/mL) (Table 1) alongside ketosis. A standard NDM genetic panel was negative. Whole-exome sequencing (WES) subsequently identified a novel homozygous variant in the *MS4A6A* gene (c.162G>C; p.Leu54Phe) (Table 2). Both parents were confirmed as heterozygous carriers (Table 3).

**Table 2:** Whole-exome sequencing (WES) findings and in silico analysis of baby #The variant is rare in the general population (gnomAD frequency: 1.2 × 10^6^). Classified as VUS due to lack of functional validation. VUS: variant of uncertain significance

Gene and Transcript	Location	Variant	Phenotype	In Silico Prediction	Clinical Significance
MS4A6A NM_022349.4	Exon 3	c.162G>C (p.Leu54Phe) (dbSNP: rs1258408007)	None reported	SIFT: Damaging PolyPhen-2: Poss. Damaging CADD: 24.5	Uncertain Significance (VUS) (PM2)#

**Table 3:** Whole-exome sequencing (WES) findings of the parents

Sample	Gene	Location	Variant	Inheritance	Remark
Mother	MS4A6A (NM_022349.4)	Exon3	c.162G>C; p.Leu54Phe (Chr11:60179951)	--	Present (Heterozygous)
Father	MS4A6A (NM_022349.4)	Exon3	c.162G>C; p.Leu54Phe (Chr11:60179951)	--	Present (Heterozygous)

Functional genetic analysis

**Figure 1:**
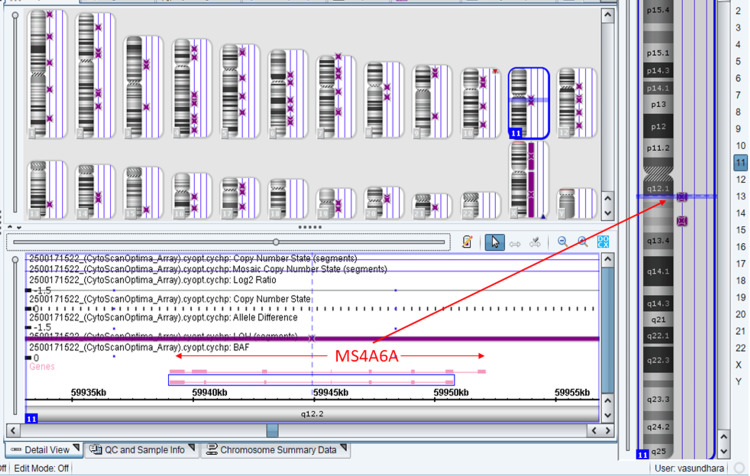
Chromosomal microarray (CMA) of baby

To validate the genomic context of the homozygous variant, a chromosomal microarray (CMA) was performed on the infant (Figure 1). The analysis showed no clinically significant deletion or duplication. However, it revealed a significant region of loss of heterozygosity (LOH) at arr[hg19] 11q12.1q12.2(59,014,657-61,457,857) hmz. This region encompasses the *MS4A6A* gene. This finding confirms that the homozygosity of the variant is due to IBD, likely resulting from shared distant ancestry between the parents, despite the lack of known consanguinity.

Allele frequency and in silico prediction

The identified variant (MS4A6A c.162G>C) is extremely rare, with an allele frequency of 1.2x10^6^ (two alleles out of 1,613,944) in the Genome Aggregation Database (gnomAD). In silico pathogenicity prediction tools suggest a deleterious effect: SIFT (Damaging), PolyPhen-2 (Possibly Damaging), and a high Combined Annotation Dependent Depletion (CADD) score (24.5), indicating high conservation and potential functional impact. Despite this, the variant is currently classified as a variant of uncertain significance (VUS) according to the American College of Medical Genetics and Genomics (ACMG) guidelines.

**Figure 2:**
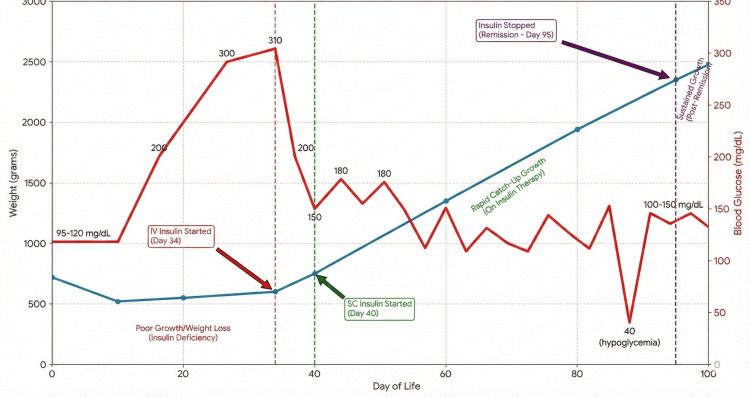
Weight gain and blood glucose level vs. day of life Graph illustrating the timeline of weight gain and blood glucose level in relation to therapeutic interventions (IV Insulin, SC Insulin) and the eventual discontinuation of therapy. SC: subcutaneous; IV: intravenous

Insulin therapy was initiated on day 34 (intravenous) and transitioned to a subcutaneous basal-bolus regimen on day 40. This led to catch-up growth, requiring weekly upward titration of the insulin dose to match the infant's rapid weight gain [12,13]. However, by day 92 (Figure 2), insulin requirements decreased, and the patient developed spontaneous hypoglycemia. Insulin was discontinued on day 95. The patient maintained euglycemia off therapy, and a repeat serum insulin level of 3.97 μIU/mL (Table 1) confirmed the restoration of endogenous secretion. Consequently, the diagnosis was revised from PNDM to TNDM [3].

## Discussion

The most critical finding of this report is the identification of a homozygous *MS4A6A* variant as a potential candidate gene for TNDM. The pathogenicity of this finding is supported by the CMA results, which identified a copy-neutral LOH region at 11q12.1q12.2 surrounding the gene. This LOH confirms that the homozygosity is authentic and driven by IBD [11], refuting the possibility of a deletion-induced hemizygosity artifact.

Proposed pathophysiology

Unlike transcription factors (e.g., PDX1, PTF1A) that cause pancreatic aplasia, MS4A6A is not known to be involved in pancreatic embryogenesis. Instead, we propose two alternative hypotheses for the transient nature of the diabetes in this patient:

Transient Islet Inflammation

MS4A6A is highly expressed in macrophages and is a key regulator of immune signalling [10]. Dysfunction of this gene may have led to a localised, transient inflammatory response within the pancreatic islets (sterile insulitis), resulting in temporary beta-cell silencing (stunning) rather than permanent destruction. As the inflammatory stimulus resolved, beta-cell function recovered.

Defective Calcium Signalling

The MS4A family of proteins shares structural homology with the CD20 calcium channel and is involved in regulating calcium flux [10]. Since insulin secretion is strictly calcium-dependent [14], a defect in *MS4A6A* could impair intracellular calcium handling in beta-cells or associated immune cells, leading to a temporary secretory failure that resolved with maturation or compensatory mechanisms.

Limitations

We acknowledge several limitations in this report. First, the identified *MS4A6A* variant is classified as a VUS (PM2), and no functional studies were performed to definitively prove pathogenicity. Second, the extreme prematurity of the infant is a confounding factor, although the clinical course was more severe than typical stress hyperglycemia [15]. Future in vitro studies are required to validate the impact of this variant on calcium flux and islet inflammation.

Management and surveillance

Management must now focus on the high lifetime risk of relapse, particularly during metabolic stress [3]. Parents have been educated on "sick day" management [16]. Long-term surveillance is mandatory, including annual HbA1c and oral glucose tolerance tests [17]. Furthermore, the heterozygous parents face a "dual-risk" profile: their carrier status combined with a polygenic family history warrants proactive metabolic surveillance [17,18]. Finally, genetic counselling should be offered, noting the current VUS status of the variant and the IBD findings [19,20].

## Conclusions

We report a case of TNDM potentially associated with a homozygous *MS4A6A* variant. Chromosomal microarray confirmed that the homozygosity was due to IBD within a region of LOH. We hypothesize that the mechanism involves transient islet inflammation or defective calcium signaling. This case identifies *MS4A6A* as a candidate gene for NDM, highlighting the need for functional validation and lifelong metabolic monitoring.
